# Recent Advances in the Electrocatalytic Performance of Nanoporous Materials for Hydrogen Evolution Reaction

**DOI:** 10.3390/nano15231782

**Published:** 2025-11-26

**Authors:** Zhangyi Li, Lin Yang, Yingqi Chen, Wence Xu, Zhonghui Gao, Jiamin Zhu, Yanqin Liang, Hui Jiang, Zhaoyang Li, Zhenduo Cui, Hao Wang, Shengli Zhu

**Affiliations:** 1 School of Materials Science and Engineering, Tianjin University, Tianjin 300350, China; 2019208063@tju.edu.cn (Z.L.); yl162022@tju.edu.cn (L.Y.); 3023208090@tju.edu.cn (Y.C.); wcxu@tju.edu.cn (W.X.); zhgao@tju.edu.cn (Z.G.); mfmzjm@tju.edu.cn (J.Z.); yqliang@tju.edu.cn (Y.L.); h.jiang@tju.edu.cn (H.J.); zyli@tju.edu.cn (Z.L.); zdcui@tju.edu.cn (Z.C.); 2 State Key Laboratory of Precious Metal Functional Materials, Tianjin University, Tianjin 300350, China; 3 Tianjin Key Laboratory of Composite and Functional Materials, Tianjin 300350, China; 4 Co-Creation Institute for Advanced Materials, Shimane University, Shimane 6908504, Japan

**Keywords:** nanoporous materials, transition metal compounds, electrocatalysis, hydrogen evolution reaction, catalyst design

## Abstract

Electrocatalytic water splitting for hydrogen production is a crucial technology in achieving carbon neutrality. The development of efficient and stable hydrogen evolution reaction (HER) electrocatalysts is a core challenge in this field. This review systematically summarizes the latest research advancements in nanoporous transition metal-based catalysts, covering metal alloys and compounds. Through strategies such as compositional optimization, crystal structure modulation, interface engineering, and nanoporous structure design, these non-precious metal catalysts exhibit outstanding performance comparable to commercial platinum-carbon catalysts across a wide pH range. This paper thoroughly discusses the catalytic mechanisms of different material systems, including electronic structure regulation, active site exposure, and mass transport optimization. Finally, the challenges faced in current research are summarized, and future directions are projected, including scalable fabrication processes and performance validation in real electrolysis cell environments. This review provides significant insights into designing next-generation efficient and stable non-precious metal electrocatalysts.

## 1. Introduction

Growing global concerns regarding the energy crisis and environmental pollution have intensified the focus on developing and utilizing clean and sustainable energy sources [1,2]. Hydrogen energy, recognized for its high energy density, zero carbon emissions during combustion, and ability to serve as a cross-seasonal and cross-regional energy carrier, is considered a foundational element of the future energy system [3,4]. Among various hydrogen production methods, electrochemical water splitting driven by renewable electricity—such as solar or wind power—represents one of the most promising routes for generating “green hydrogen,” enabling a clean transition from “green electricity” to “green hydrogen” [5,6,7].

The water electrolysis process consists of two key half-reactions: the oxygen evolution reaction (OER) at the anode and the HER at the cathode [8,9]. Hydrogen evolution reaction kinetics are inherently slow in alkaline media requiring highly efficient electrocatalysts to reduce the overpotential, minimize energy losses, and improve hydrogen production efficiency [10,11,12]. Although platinum (Pt)-based materials are currently the most effective HER catalysts, their high cost and scarcity limit large-scale industrial application. Therefore, developing non-precious metal or low-precious-metal-content HER catalysts that exhibit high activity, strong durability, and low cost is essential for the commercialization of water electrolysis technology [13].

In recent years, nanoporous materials have attracted considerable interest in catalysis owing to their distinctive structural features [14,15]. These materials typically offer high specific surface area, abundant porous networks, and adjustable pore size distributions, which provide several benefits as electrocatalysts. Moreover, innovative designs enable the creation of “single-atom catalysts,” where active sites are isolated and anchored on a conductive porous framework, maximizing atom utilization efficiency and catalytic performance [16,17,18].

**Figure 1:**
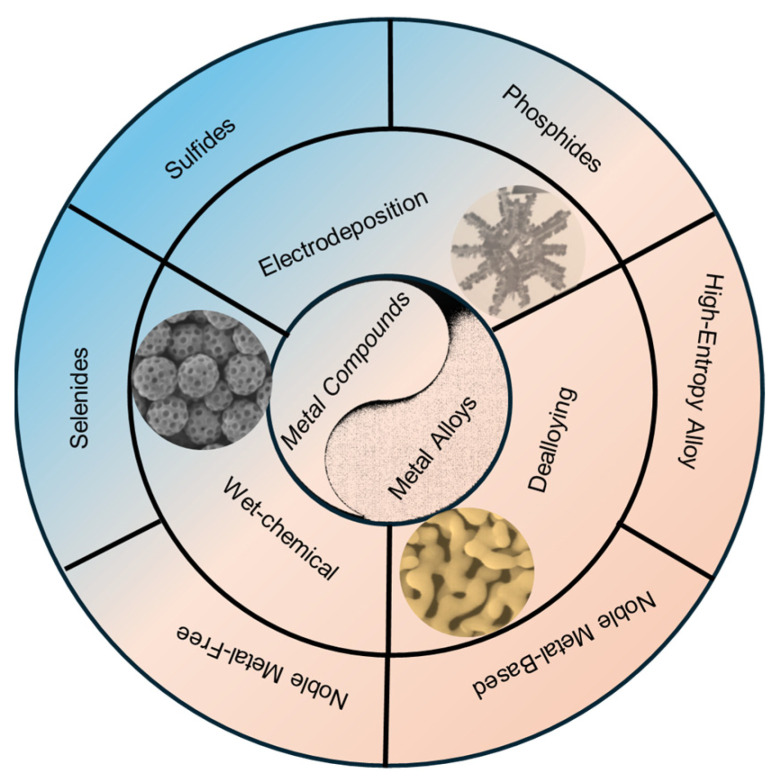
Classification of metal-based materials and synthesis methods for advanced catalysts.

The field of nanoporous electrocatalysts for the HER is advancing rapidly, with many reviews focusing on specific material classes such as alloys, phosphides, or sulfides. However, a complementary and unified perspective is essential for further progress, one that examines these materials through cross-scale design principles. This approach integrates atomic-level modifications, nanoscale structural engineering, and macroscale electrode architecture to understand how these factors collectively influence HER performance under industrially relevant conditions. This review adopts this integrative philosophy, providing a comprehensive summary of recent progress in nanoporous materials for the HER. It first explains the fundamental mechanisms of the HER and key performance evaluation parameters. It then categorizes and examines various types of nanoporous materials, including metallic alloys and metal compounds, addressing their design strategies, synthesis methods, and HER catalytic performance (Figure 1). Special attention is given to frontier systems, such as high-entropy alloys, single-atom catalysts on porous scaffolds, and heterostructures formed between different metal compounds. By framing recent advances through the lens of synergistic interactions between composition, porosity, and interface, this review highlights how these cross-scale strategies can guide the rational design of next-generation nanoporous HER electrocatalysts. The review also explores the role of porous structures in enhancing catalytic activity, stability, and mass transport and concludes with a discussion of current challenges and future research directions to develop efficient, durable, and scalable electrocatalysts for water electrolysis.

## 2. Hydrogen Evolution Reaction Mechanisms and Performance Characterization

### 2.1. Fundamental Mechanisms of the Hydrogen Evolution Reaction

The hydrogen evolution reaction is a multi-step electrochemical process influenced by the electrolyte environment and catalyst properties. In acidic conditions, the reaction typically follows the Volmer–Heyrovsky or Volmer–Tafel pathway. The Volmer step entails the electrochemical adsorption of a proton to form an adsorbed hydrogen intermediate (H_3_O^+^ + * + e^−^ → H* + H_2_O). This is followed by the Heyrovsky step (H* + H_3_O^+^ + e^−^ → H_2_ + H_2_O) or the Tafel step (H* + H* → H_2_) to produce hydrogen molecules [19]. In alkaline media, the HER mechanism involves an initial water dissociation step (H_2_O + * + e^−^ → H* + OH^−^), which introduces an additional energy barrier. As a result, HER kinetics are generally slower in alkaline environments compared to acidic conditions [20].

Theoretical research has identified the hydrogen ΔG_H_* as a critical descriptor of HER activity. An optimal catalyst should have a ΔG_H_* value near zero, balancing hydrogen intermediate adsorption and desorption. This principle has informed the development of HER catalysts through strategies such as alloying, heteroatom doping, and defect engineering to adjust electronic structures and optimize ΔG_H_* values [10,21].

### 2.2. Performance Characterization Methods and Metrics System

A thorough evaluation of HER catalyst performance involves multiple electrochemical techniques to form a complete assessment system. Activity is mainly evaluated using polarization curves, with key metrics including the onset overpotential and the overpotential at specific current densities. Kinetic properties are assessed through the Tafel slope and exchange current density (j_0_), where the Tafel slope indicates the rate-determining step, and j_0_ reflects the catalyst’s intrinsic activity.

Stability tests typically involve cyclic voltammetry and chronoamperometry/chronopotentiometry measurements. Cyclic voltammetry assesses structural stability over numerous potential cycles, while chronoamperometry/chronopotentiometry tests durability under constant overpotential or current density. Post-stability analysis uses microstructural and surface characterization techniques to examine degradation mechanisms.

The electrochemical active surface area (ECSA), measured via the double-layer capacitance method, allows for comparison of intrinsic activities between catalysts. Electrochemical impedance spectroscopy (EIS) helps identify interfacial reaction resistances, providing details on charge transfer resistance.

### 2.3. Performance Enhancement Mechanisms of Nanoporous Structures

Recent advances in nanoporous hydrogen evolution electrodes show that precise control over pore size distribution and channel connectivity can optimize transport pathways for reactants and products, reducing mass transfer resistance and concentration polarization [22,23,24]. In the alkaline HER, porous structures not only increase the number of active sites but also promote water molecule transport and dissociation, mitigating inherent kinetic limitations. Additionally, continuous porous conductive frameworks facilitate efficient electron transport, enabling rapid charge transfer at reaction interfaces [25,26,27].

## 3. Design and Preparation of Nanoporous Electrodes

The preparation of transition metal-based nanoporous catalysts employs a range of material synthesis strategies to achieve precise control over pore structure, active site distribution, and electronic properties. Established methods like dealloying enable the formation of three-dimensional bicontinuous porous networks by selectively dissolving more active components from precursor alloys yielding mechanically strong, highly conductive frameworks [28,29,30,31]. Meanwhile, wet-chemical methods such as hydrothermal and solvothermal synthesis facilitate the direct growth of nanostructured porous materials like nanosheet or nanowire arrays on conductive substrates [32]. Electrodeposition provides another flexible approach for producing binder-free electrodes with customized porosity and strong substrate adhesion [33,34].

Dealloying is a commonly used top-down method for creating nanoporous metallic electrodes. It involves selectively dissolving the more active element from a precursor alloy, resulting in a porous structure composed of the more noble metal [35,36,37,38]. The resulting material exhibits high electrical conductivity, excellent pore connectivity, and strong mechanical stability—properties essential for withstanding vigorous gas evolution and long-term operation in electrocatalytic reactions [25]. Morphological features such as pore size, ligament diameter, and specific surface area can be finely adjusted by modifying the precursor alloy composition, etching parameters, and post-treatment conditions [18,39].

Wet-chemical synthesis methods, particularly hydrothermal and solvothermal techniques, provide an adaptable and scalable way to directly fabricate nanostructured porous electrodes on conductive substrates. These solution-based processes involve chemical reactions in sealed vessels at high temperatures and pressures, allowing for precise control over crystal nucleation and growth [40,41,42]. Hydrothermal methods use aqueous solutions, while solvothermal synthesis employs non-aqueous solvents, often enabling better control over morphology and crystallinity. A major advantage of these methods is their ability to directly grow oriented nanostructures—such as vertically aligned nanosheet arrays, interconnected nanowire networks, or hierarchical microspheres—on various current collectors like nickel foam, carbon cloth, or titanium mesh [41,43,44,45]. Direct growth eliminates the need for polymer binders and conductive additives, ensuring good electrical contact, strong mechanical adhesion, and structural integrity. Porosity can be tailored through self-assembly, oriented attachment, or structure-directing agents. For instance, three-dimensional porous networks of transition metal phosphide nanowires or interconnected sulfide nanosheets can be easily synthesized [46,47].

Electrodeposition is a highly controllable and scalable electrochemical technique for directly fabricating binder-free, porous catalytic electrodes. This method involves reducing metal ions from an electrolyte solution onto a conductive substrate under an applied potential or current, allowing for precise control over nucleation, growth, and final morphology [48,49]. A significant advantage of electrodeposition is its ability to produce adherent, porous coatings with strong mechanical and electrical connection to the substrate, avoiding the use of polymer binders that can block active sites and increase interfacial resistance [50,51,52]. Porosity can be designed through various strategies, such as dynamic bubble templating, where hydrogen or oxygen bubbles generated during deposition act as temporary templates for creating open macroporous structures [53]. Alternatively, electrodeposition of nanostructured alloys followed by selective dealloying, or co-deposition of composites with sacrificial elements, can yield high-surface-area architectures with tunable pore size distributions [54]. This allows for the fabrication of diverse nanostructures, including porous foams, dendritic networks, nanowire arrays, and nanosheet assemblies, directly on complex or flexible substrates.

Based on a comprehensive evaluation of scalability, environmental impact, and economic viability, electrodeposition emerges as the most promising method for industrial-scale production of nanoporous HER catalysts, due to its compatibility with continuous manufacturing processes, ambient operational conditions, and capability for direct fabrication of binder-free electrodes. While green dealloying variants (e.g., liquid metal or vapor phase dealloying) show potential for creating structurally robust porous frameworks, they require further development to address challenges related to precursor cost and waste management. Other methods, including hydrothermal synthesis and laser-based processing, remain limited by batch-processing constraints or low throughput. Ultimately, successful scale-up will require combining these syntheses with techno-economic analysis and validation under realistic electrolyzer operating conditions.

## 4. Nanoporous Electrocatalysts for HER

Synthesis methodologies offer the fundamental capabilities and diversity essential for designing material systems, while different material systems utilize these methods to realize distinct structures and functions. This synergistic interplay lies at the heart of research on nanoporous HER electrocatalysts. By precisely adjusting process parameters across various synthesis strategies, researchers gain accurate control over pore size, chemical composition of pore walls, and three-dimensional interconnectivity while also tailoring structures to fulfill the specific requirements of different material systems. The tight integration of synthesis techniques and material systems permits the rational selection and structural optimization of suitable materials based on target application scenarios and performance needs, ultimately contributing to the synergistic improvement of catalytic activity, stability, and cost efficiency. It is this dynamic interaction between progress in fabrication techniques and innovations in materials that drives the advancement of nanoporous HER electrocatalysts.

### 4.1. Transition Metal Alloys

Transition metal alloys offer a highly adaptable platform for designing high-performance HER electrocatalysts by leveraging electronic modulation and synergistic effects among multiple metallic elements. In contrast to single-metal systems, alloying permits effective tuning of the electronic structure of active sites and optimization of ΔG*_H_, leading to a marked increase in intrinsic catalytic activity [55,56,57,58,59]. The incorporation of nanoporous structures further augments the catalytic performance of transition metal alloys. Their three-dimensionally interconnected pore channels not only greatly expand the active specific surface area but also promote mass transport and gas release during reactions [35,60,61]. Current research emphasizes systems such as noble metal-free alloys [10,62,63,64,65], noble metal-based systems [66,67,68], and multi-component HEAs [69,70,71,72,73,74]. Advanced synthesis approaches enable precise control over composition, pore architecture, and surface properties, accelerating the development of water electrolysis technology toward greater efficiency, durability, and affordability.

#### 4.1.1. Noble Metal-Free Systems

Alloy systems based on non-precious metals like nickel, cobalt, iron, molybdenum, and tungsten have attracted considerable research interest as alternatives to precious metal catalysts, owing to their low cost and Earth abundance [75,76,77,78,79,80]. Through careful adjustment of alloy composition and nanoporous architecture, these materials achieve catalytic activity in alkaline media that rivals that precious metals.

Compositional optimization is a fundamental strategy for enhancing the performance of nanoporous alloy catalysts. In the Ni-Fe-Mo ternary alloy, the incorporation of Fe lowers the d-band center of Ni, moderately weakening hydrogen adsorption strength, while Mo significantly enhances the water dissociation process. This multi-element synergistic effect enables the optimized Ni/NiFeMoOx@Ni foam alloy to achieve a current density of 10 mA cm^−2^ at an overpotential of only 22 mV under alkaline conditions, significantly outperforming binary alloy counterparts [81]. To advance the development of cost-effective and efficient electrocatalysts for overall water splitting, Liu et al. successfully fabricated a self-supported Ni_18_Fe_12_Al_70_ multi-element alloy system using an innovative laser-assisted aluminum doping strategy [82]. The introduction of aluminum effectively modulated the material’s morphology, composition, and phase structure, leading to the formation of a unique hybrid architecture comprising Ni_2_Al_3_ and Ni_3_Fe phases. Benefiting from this multi-phase synergistic effect, the catalyst demonstrated remarkable bifunctional electrocatalytic activity and stability. It required overpotentials of only 188 mV for the HER to achieve a current density of 100 mA cm^−2^ and maintained stable operation for over 100 h under overall water-splitting conditions. Theoretical calculations provided further insight, confirming that the synergistic interaction between the Ni_2_Al_3_ and Ni_3_Fe phases optimizes the adsorption behavior of reaction intermediates, thereby cooperatively enhancing the overall catalytic kinetics.

Structural design offers another key approach to enhancing catalytic performance. Hierarchical porous structures increase the electrochemical active surface area, facilitating the mass transport of electrolytes, reactants, and bubbles, thus effectively suppressing concentration polarization and ohmic losses. This multiscale pore structure can be constructed in metallic systems through alloying or templating methods, creating multiscale (macro–meso–micro) channels that form a bicontinuous framework with rapid transport pathways and abundant exposed sites, thereby improving catalytic activity [83,84,85,86]. For example, in Ni-Mo hydrogen evolution electrodes, a templated growth method successfully created an ordered porous NiMo film that can sustain industrial-level currents [87,88,89]. In 1 M KOH, the 3D ordered macroporous NiMo electrode achieved current densities of 500 mA cm^−2^ and 1000 mA cm^−2^ at overpotentials of 306 mV and 491 mV, respectively, while maintaining excellent stability at high current densities. The mechanism behind this improvement lies in the fine-tuning of the pore structure to optimize bubble release and electrolyte transport, thereby enhancing electrocatalytic efficiency. Similarly, the Fe_2_Mo/Cu nanoporous alloy catalyst increases the catalyst’s surface area and active sites by incorporating Fe_2_Mo nanoparticles onto a porous copper framework, enhancing charge transfer rates, and thereby improving catalytic activity and stability. In 1 M KOH, this catalyst achieves a current density of 1 A/cm^2^ at an overpotential of 200 mV, and the current density remains stable for over 400 h of operation [90]. The performance improvement primarily arises from the incorporation of Fe_2_Mo particles, which adjust the alloy’s electronic structure to optimize hydrogen adsorption and dissociation, while the porous copper framework further enhances charge transfer efficiency and catalytic activity.

**Figure 2:**
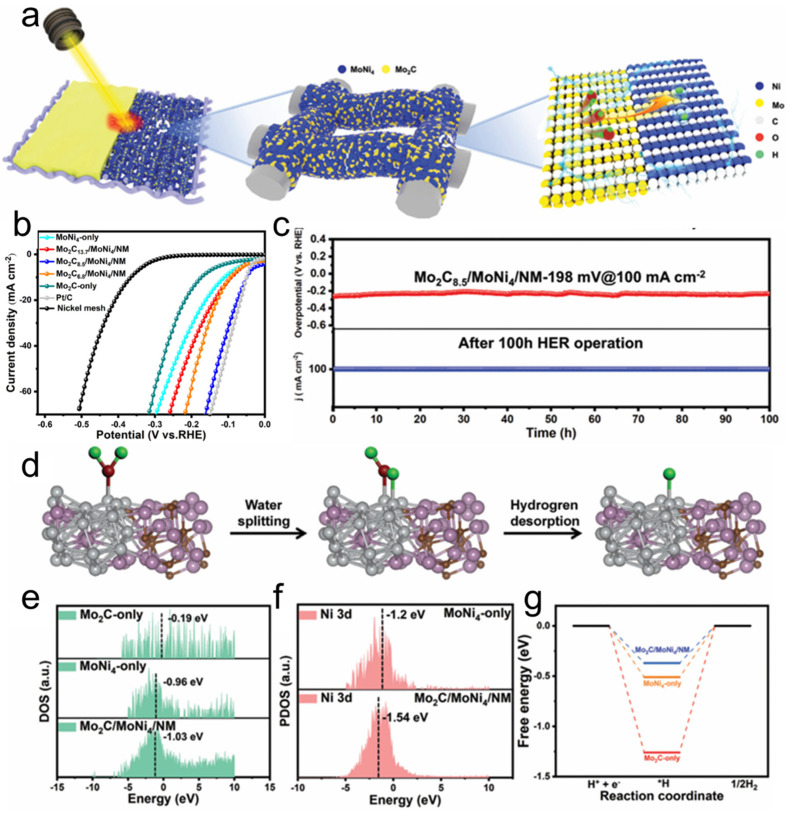
(**a**) Schematic illustration of the formation of Mo_2_Cx/MoNi_4_/NM heterojunction electrode; (**b**) Linear sweep voltammetry (LSV) curves; (**c**) Chronopotentiometric curve of water electrolysis for Mo_2_C_8_._5_/MoNi_4_/NM electrode serving as both cathode and anode under a constant current density of 100 mA cm^−2^ for 100 h. (**d**) Schematic illustration of the proposed reaction mechanism on Mo_2_C/MoNi_4_/NM (gray, blue, brown, red, and green spheres representing Ni, Mo, C, O, and H atoms), Mo_2_C (along the (101) plane) embedded into the periodic MoNi_4_ (121) plane was selected as the exposed crystal plane in the selected electron diffraction (SAED) mode. (**e**) Total DOS of Mo_2_C-noly, MoNi_4_-noly and Mo_2_C/MoNi_4_/NM. (**f**) Calculated PDOS of different catalysts with d band center positions of Ni active sites. (**g**) Gibbs free energy profiles for hydrogen adsorption (ΔG_H_*) on MoNi_4_-only, Mo_2_C-only, and Mo_2_C/MoNi_4_/NM [93].

The combination of heterointerface engineering and dual-active-site design represents a cutting-edge approach for developing advanced nanoporous alloy catalysts for alkaline HER. Recent advances demonstrate that integrating these strategies can overcome inherent limitations of single-metal systems [91]. For example, a fault-rich heterostructure Mg-Ni alloy synthesized via molten salt electrodeposition illustrates how non-equilibrium processing can reconcile incompatible thermodynamic demands between phase boundaries and stacking faults [92]. This catalyst delivers outstanding performance with an overpotential of 69.2 mV at 10 mA cm^−2^ and remarkable stability, maintaining 600 mA cm^−2^ for 38 h, outperforming commercial Pt catalysts. The improvement stems from synergistic effects: heterointerfaces promote electron transfer and optimize intermediate adsorption/desorption, while stacking faults introduce lattice strain that accelerates proton transfer. Zhao et al. developed a facile strategy to fabricate a high-performance HER electrode. In this approach, homogeneous ink was prepared by blending Mo and Ni metal powders with an organic solvent [93]. This ink was subsequently doctor-bladed onto a nickel mesh substrate and processed via laser scanning (Figure 2a). The organic solvent, acting as a volatile carbon source, reacted with Mo powder under high-energy laser irradiation to form Mo_2_C. Concurrently, the localized high-temperature and high-pressure environment facilitated the reaction between Mo and Ni, leading to the formation of a primary MoNi_4_ phase. As shown in Figure 2b, the process yielded an optimal Mo_2_C/MoNi_4_/NM heterostructure. Electrochemical measurements in 1.0 M KOH demonstrated that the optimized Mo_2_C_8.5_/MoNi_4_/NM electrode required an overpotential of only 49.5 mV to achieve a current density of 10 mA cm^−2^. This remarkable HER performance is attributed to the three-dimensional nanoporous architecture and the synergistic effects between the trace amounts of Mo_2_C and the MoNi_4_ phase. Furthermore, Figure 2c confirms that the electrode exhibited excellent operational stability, maintaining its performance for over 100 h at a high current density of 100 mA cm^−2^. Combined experimental characterization and theoretical calculations revealed that the constructed Mo_2_C/MoNi_4_ heterostructure plays a dual role: it significantly enhances electrical conductivity and charge transfer kinetics. Moreover, it optimizes the electronic structure of Ni by downshifting its d-band center. This electronic modulation effectively weakens the hydrogen intermediate (H*) adsorption and promotes H_2_ desorption, thereby accelerating the overall HER kinetics (Figure 2d–g).

#### 4.1.2. Noble Metal-Based Systems

Incorporating small amounts of precious metals such as platinum, palladium, or ruthenium into transition metal matrices optimizes electronic structure through strain and ligand effects, significantly boosting intrinsic catalytic activity [94,95,96,97]. These materials retain high activity while maximizing noble metal atom utilization via nanoporous structures, offering a viable route to “low precious metal content, high performance.”

The addition of trace amounts (typically below 5 at.%) of noble metals like Pt, Pd, or Ru into transition metal matrices (e.g., Ni or Co) enables the formation of gradient-distributed Pt-skin structures or noble metal-enriched near-surface regions. These configurations maximize noble metal atomic utilization and allow for fine-tuning of the d-band center of active sites through ligand and strain effects, optimizing the adsorption energy of reaction intermediates [97,98,99]. For example, a Ni–Pt alloy with 1.5 at.% Pt exhibits mass activity 3.2 times higher than commercial Pt/C catalysts, mainly due to compressive strain lowering the d-band center and optimizing hydrogen adsorption free energy. Introducing a third or fourth transition metal element (e.g., Cu, Mo or W) to form multi-component alloys can induce more complex electronic synergy, enabling finer control over catalytic performance [78,100].

**Figure 3:**
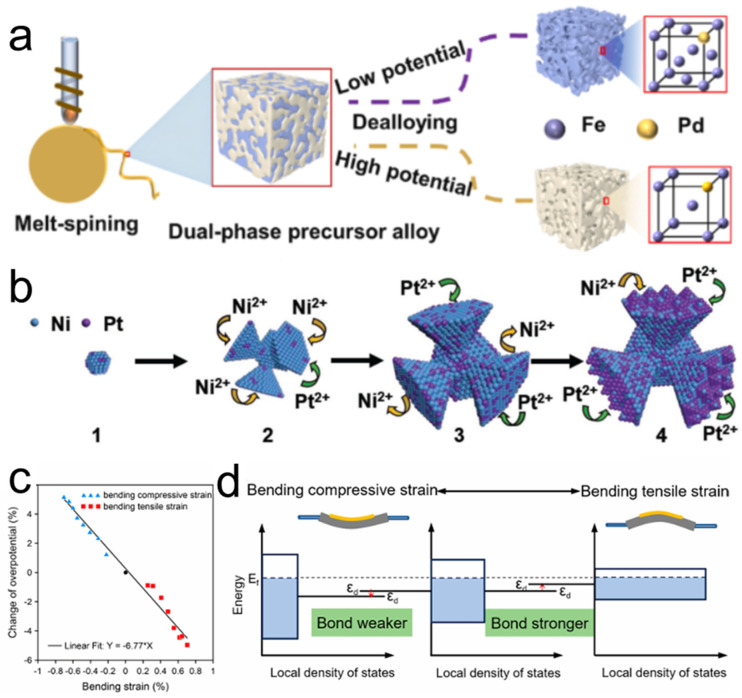
(**a**) Schematic illustration of Fe based nanopores with adjustable phase formed through electrochemical dealloying strategy [101]. (**b**) Schematic illustration of the lotus-thalamus-shaped Pt-Ni alloy superstructures [102]. (**c**) the bending strain dependence of HER overpotentials at the current density of 5 mA/cm; and (**d**) schematic illustration of d-band center changes with bending. The compressive strains shift the d-band center downward, resulting in weaker binding to reaction intermediates while the tensile strains lead to the opposite results of upward shift in the d-band center and strengthened binding with reactants [14].

Tailoring the crystalline phase of catalysts can markedly influence their catalytic properties. Recent studies show that metastable or unconventional crystal structures often exhibit superior catalytic activity compared to conventional phases. For instance, face-centered cubic (fcc) Fe–Pd nanoparticles show significantly better alkaline HER performance than their body-centered cubic (bcc) counterparts, due to an optimized d-band center in the fcc structure that weakens metal–hydrogen interactions and lowers reaction energy barriers (Figure 3a). Additionally, creating heterophase interfaces (e.g., metal/intermetallic heterojunctions) can induce strong interfacial electronic interactions, further optimizing intermediate adsorption behavior [101]. A one-pot solvothermal approach can yield well-crystalline, lotus-thalamus-shaped Pt-Ni alloy superstructures (ASs) featuring Pt-rich surfaces and a mixed hcp/fcc crystal phase (Figure 3b). The formation mechanism involves a transformation from initial Ni-rich polyhedrons to branched nanostructures, followed by a galvanic replacement reaction and a facet-selective codeposition process. This intricate growth pathway culminates in a catalyst with superior electrocatalytic performance for the alkaline HER, surpassing the activity and stability of commercial Pt/C. Consequently, this work establishes a synthetic paradigm for creating spatially heterogeneous nanomaterials, underscoring their significant potential for application in diverse catalytic reactions [102].

Macroscopic strain engineering has been established as an effective strategy for modulating the catalytic properties of dealloyed nanoporous catalysts. As illustrated in Figure 3c, experimental evidence reveals a linear correlation between applied strain and HER overpotential within the elastic regime, with each 0.1% of compressive or tensile strain inducing a change in hydrogen binding energy of approximately 2.9 eV. Quantitative assessments further demonstrate that compressive strains generally impair HER activity, whereas tensile strains enhance the catalytic performance of NPG. These observations align well with predictions from the d-band center model (Figure 3d): when strain induces a shift in the d-band center toward an energy range more favorable for reactant adsorption, catalytic activity is improved. Conversely, a shift away from the optimal energy range raises the reaction activation barrier, thereby diminishing catalytic efficiency [14].

While most studies on noble metal-based nanoporous catalysts focus on the alkaline HER, their application in acidic media, particularly for proton exchange membrane water electrolysis (PEMWE), is of great importance due to higher proton conductivity and faster kinetics. However, acidic environments pose severe durability challenges due to catalyst dissolution under open-circuit potential and dynamic operation. Recently, Li et al. [103] demonstrated a dynamic dissolution-deposition equilibrium mechanism to achieve unprecedented HER stability in acidic PEMWE. By dealloying a FeCoNiNbPt high-entropy alloy, they fabricated a self-supported nanoporous catalyst with a hierarchical porous structure. The catalyst features dual-functional components: an amorphous NbOx buffer layer that suppresses metal dissolution and multicomponent Pt_3_(FeCoNi) nanocrystals that synergistically enhance HER activity. In 0.5 M H_2_SO_4_, the optimized catalyst exhibits an overpotential of 137 mV at 1 A cm^−2^ and a Tafel slope of 39.3 mV dec^−1^, outperforming commercial Pt/C. Remarkably, it maintains stable operation for over 2200 h at 1 A cm^−2^ (including intermittent operation) and withstands 1,000,000 cycles with less than 2% activity decay. The exceptional durability is attributed to the dynamic equilibrium between dissolution and deposition, where the NbOx layer stabilizes the surface and the high-entropy effect minimizes atomic diffusion. Moreover, the catalyst has a low Pt loading of 8.87 wt.% (60% reduction compared to Pt/C) and shows excellent performance in a PEMWE device, operating steadily for 1000 h at high current densities (2–3 A cm^−2^). This work highlights the potential of nanoporous high-entropy alloys with protective layers for achieving both high activity and durability in the acidic HER, providing a new design strategy for industrial PEMWE applications.

In parallel to enhancing stability for the HER alone, the development of bifunctional catalysts capable of efficiently catalyzing both the HER and the OER is considered important for simplifying PEMWE system architecture and reducing costs. In this direction, Shi et al. [104] reported a nanoporous PdIr bifunctional alloy catalyst that demonstrates notable overall water-splitting performance in acidic media. The nanoporous Pd_50_Ir_50_ (np-Pd_50_Ir_50_) prepared by dealloying possesses a three-dimensional interconnected hierarchical pore structure. XPS and XAS analyses confirmed strong electronic coupling between Pd and Ir atoms, with electron transfer from Ir to Pd. This synergistic electronic modulation resulted in Ir sites with a higher oxidation state and a downshift in the Pd d-band center. These changes are understood to optimize the adsorption energy of OER intermediates on Ir sites and weaken the adsorption strength of HER intermediates on Pd sites. In 0.5 M H_2_SO_4_ electrolyte, np-Pd_50_Ir_50_ required an overpotential of only 20 mV to achieve 10 mA cm^−2^ for HER, outperforming commercial Pt/C (25 mV), and exhibited a Tafel slope of 24 mV dec^−1^, suggesting faster reaction kinetics. Concurrently, the catalyst demonstrated excellent OER performance, requiring an overpotential of 217 mV to reach 10 mA cm^−2^, which is substantially lower than that of commercial Ir/C (300 mV). Furthermore, when np-Pd_50_Ir_50_ was employed as both the cathode and anode in a full water electrolyzer, a cell voltage of only 1.52 V was required to achieve 10 mA cm^−2^. This performance is 80 mV lower than the benchmark Pt/C||Ir/C couple (1.60 V) and was maintained with good stability. Theoretical calculations provided insights into the origin of the bifunctional activity, indicating that alloying Pd with Ir modulates the d-band center of Ir sites, lowering the energy barrier for the potential-determining step in the OER (*OOH → O_2_). Meanwhile, the downshift in the Pd d-band center optimizes the hydrogen adsorption free energy for HER.

#### 4.1.3. High-Entropy Alloy Systems

As an emerging material system composed of five or more principal elements in equimolar or near-equimolar ratios, HEAs exhibit unique physicochemical properties and considerable catalytic potential due to high entropy, lattice distortion, and “cocktail” effects. Breaking from traditional alloy design concepts, the multi-principal-element nature of HEAs creates numerous active sites with diverse energy distributions on the material surface, offering more opportunities to optimize the adsorption of reaction intermediates [63,70,84,105]. Research shows that some HEA systems demonstrate catalytic activity close to platinum in both acidic and alkaline environments, along with excellent structural stability, indicating promising directions for next-generation high-efficiency HER catalysts [106,107].

Recent years have seen considerable progress in applying HEAs to the electrocatalytic HER [71,108]. Their superior performance largely stems from tunable multi-component compositions and unique structural features, which collectively optimize electronic structures and reaction pathways. For instance, a nanoporous FeCoNiCuTi HEA prepared via arc melting and dealloying showed exceptional catalytic performance in alkaline medium (1.0 M KOH), requiring an overpotential of only 134 mV to reach 1 A cm^−2^ and exhibiting a Tafel slope of 43 mV dec^−1^, outperforming commercial Pt/C catalysts [70]. This outstanding activity is attributed to synergistic effects among multiple elements: lattice distortion induces local strain fields that modify the electronic structure of key active sites (e.g., Co and Ni). Meanwhile, the surface HEA provides multiple active sites and leverages the distinct adsorption behaviors of *H and *OH intermediates on NiFeCoCu and Ti components, thereby accelerating water dissociation and facilitating the adsorption/desorption of *H as well as H_2_ recombination. Benefiting from a three-dimensional interconnected columnar nanoporous structure that promotes electron transfer and mass transport of the electrolyte, the self-supported NiFeCoCuTi electrode demonstrates outstanding alkaline HER performance, promoting water molecule cleavage in the Volmer step and improving alkaline HER kinetics.

**Figure 4:**
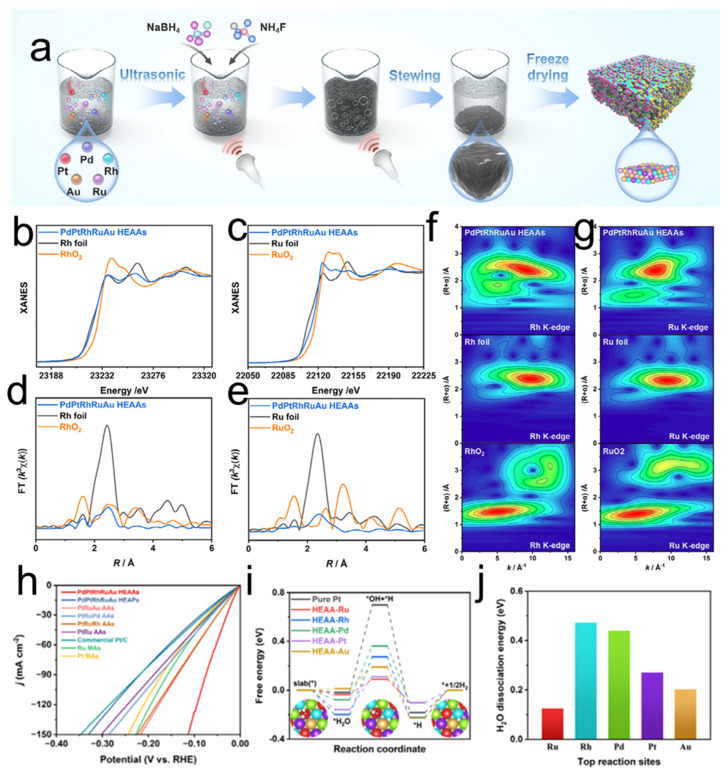
(**a**) Schematic illustration of the preparation process of PdPtRhRuAu HEAAs. (**b**,**c**) Rh K-edge XANES and Ru K-edge XANES spectrum of PdPtRhRuAu HEAAs; (**d**,**e**) Fourier transform EXAFS spectra of PdPtRhRuAu HEAAs and the references Rh K-edge and Ru K-edge; (**f**,**g**) WT-EXAFS contour plots of Rh K-edge and Ru K-edge for PdPtRhRuAu HEAAs; (**h**) The LSV curves; (**i**) the calculated free energies of HER on different active sites under non-acidic condition; (**j**) H_2_O dissociation energies on representative active sites [109].

In a parallel development that underscores the versatility of advanced material design, Liu et al. demonstrated a complementary strategy by constructing multicomponent high-entropy alloy aerogels (HEAAs) via a facile ultrasound-assisted approach [109]. Figure 4a schematically illustrates this synthetic route, where an ultrasonic field drives the simultaneous co-reduction and self-assembly of up to five distinct metal precursors (Pd, Pt, Rh, Ru, Au) into a three-dimensional porous aerogel network. Critical evidence for the successful formation of a homogenous solid-solution phase and the crucial unsaturated coordination sites was provided by synchrotron-based X-ray absorption fine structure (XAFS) spectroscopy. As shown in Figure 4b–g, the X-ray absorption near edge structure (XANES) spectra confirmed the metallic state of the constituent elements, while the extended X-ray absorption fine structure (EXAFS) R-space spectra and their corresponding fitting parameters revealed a significant reduction in the coordination numbers of Rh and Ru compared to their pure metal foils. This directly indicates the presence of low-coordination, unsaturated sites induced by the porous aerogel matrix and lattice distortion effects inherent to HEAs. The electrocatalytic superiority of this structure was further elucidated by theoretical calculations. In alkaline medium (1 M KOH), the PdPtRhRuAu HEAAs achieve an ultralow overpotential of 12 mV at 10 mA cm^−2^ and a Tafel slope of 17.02 mV dec^−1^, outperforming commercial Pt/C catalysts while maintaining remarkable stability over 160 h of continuous operation (Figure 4h). The established structural model and the corresponding free energy diagrams for the water dissociation step (*H_2_O → *H + *OH) under alkaline conditions (Figure 4i,j) revealed that the synergistic interplay of the multi-element active sites, particularly the Ru sites, drastically reduces the energy barrier for the Volmer step—the rate-determining step in alkaline HER. This work collectively highlights that, in contrast to the built-in electric field mechanism in 2D/3D heterojunctions, the catalytic enhancement in HEAAs stems from the synergistic combination of a highly porous network that ensures efficient mass transport and the creation of abundant, electronically optimized active sites through the high-entropy effect, offering a distinct pathway for engineering high-performance, pH-universal electrocatalysts.

While such compositional engineering in crystalline HEAs is fruitful, significant attention has also been directed toward manipulating their amorphous counterparts. As exemplified by Liu et al., who designed a high-entropy amorphous alloy with endogenous nanoscale phase separation, a fully amorphous nanoporous catalyst was first obtained via selective phase corrosion [110]. Subsequent controlled etching further constructed a unique amorphous/crystalline heterostructure, where embedded nanocrystalline flakes induce significant lattice distortion at the interfaces to generate a high density of active sites. Moreover, this heterostructure enables directional charge transfer and downshifts the d-band center, collectively optimizing the adsorption/desorption behavior of intermediates. This strategic design yielded exceptional bifunctional activity for overall water splitting, as demonstrated by the AC-NP-CuNiCo catalyst, which required a low cell voltage of only 1.53 V to achieve a current density of 10 mA cm^−2^ in alkaline electrolyte.

Besides liquid metal dealloying (LMD), vapor phase dealloying (VPD) is another emerging and environmentally friendly method suitable for fabricating nanoporous HEAs [111]. VPD exploits differences in saturated vapor pressures among metallic elements. Under vacuum or inert atmosphere at elevated temperatures, elements with higher vapor pressure in the HEA precursor sublimate and evaporate selectively, while components with lower vapor pressure remain and reorganize into a nanoporous structure. This technique avoids corrosive chemical solutions and extensive post-processing, making it a greener alternative to conventional dealloying [112,113]. For example, VPD has been successfully used to fabricate nanoporous HEAs such as ZnCoCrFeNi-based systems, where highly volatile elements (e.g., Zn or Mg introduced as sacrificial components) are selectively removed, yielding a porous high-entropy scaffold with high specific surface area and tunable composition. The resulting nanoporous HEAs show promising electrocatalytic properties, including enhanced activity and durability for reactions like the OER. The phase evolution and pore formation mechanisms in VPD-processed HEAs are complex, involving diffusion-driven reorganization and surface segregation under thermal treatment. Studies suggest that elemental vacancy diffusion barriers and interfacial energy minimization govern the final nanoporous morphology, with certain low-index crystal facets facilitating preferential sublimation and pore nucleation. These insights are crucial for optimizing VPD parameters to achieve tailored nanoporous structures in multicomponent HEAs [114,115].

While HEAs have demonstrated remarkable potential for the alkaline HER, their application in acidic media, particularly for PEMWEs, is also attracting increasing attention. The extremely corrosive acidic environment imposes stringent demands on the corrosion tolerance of catalysts. The multi-principal element characteristics and unique local chemical environments of HEAs offer new possibilities for designing acidic HER catalysts that combine high activity with robust stability. A notable example is the recent development of a nanoporous HEA, denoted as HEA8 (composition: AlAuIrNbPtRhRuTa), guided by an “inverse analysis” strategy, which demonstrated outstanding performance in overall water splitting under acidic conditions [116]. This innovative approach involved initially immersing nanoporous ultra-HEA containing 23 elements in an acidic environment. By analyzing the elements that remained stable in the residue, the key components resistant to acidic corrosion were identified retrospectively. This informed the precise synthesis of the HEA8 catalyst. In 0.5 M H_2_SO_4_ electrolyte, HEA8 required an overpotential of only 41 mV to achieve a current density of 10 mA cm^−2^ for the HER, a performance comparable to commercial Pt/C (37 mV). More importantly, when configured in a symmetric HEA8||HEA8 electrolyzer for overall water splitting, it required a low cell voltage of only 1.51 V to reach 10 mA cm^−2^ and retained approximately 91% of its initial activity over a 30 h test, outperforming the benchmark IrO_2_||Pt/C couple. This study not only presents a high-performance bifunctional HEA catalyst for acidic HER/OER but also provides an insightful design strategy via inverse analysis for developing new HEAs tailored for harsh operating environments. However, it must be acknowledged that significant challenges remain before HEA catalysts can be practically deployed in PEM electrolyzers. For instance, in the study, the long-term stability of HEA8 as the anode in a PEMWE configuration still lagged that of commercial IrO_2_. This indicates that the leaching and reconstruction behavior of surface elements in HEAs under the highly oxidizing, high-potential conditions at the anode require more in-depth investigation. Furthermore, most reported HEAs exhibiting excellent acidic HER performance still contains a considerable proportion of precious metals, even if the total loading is optimized. A critical direction for future research lies in further reducing the precious metal content or exploring fully non-precious metal, acid-tolerant HEA systems to enable their scalable application.

The practical deployment of high-entropy alloys requires balancing their technical advantages against environmental and economic considerations. While HEAs’ corrosion resistance and potential use of earth-abundant elements may improve sustainability through extended device lifetimes and reduced critical material dependence, these benefits are offset by energy-intensive synthesis and complex recycling challenges. Economically, high production costs from premium precursors and specialized processing currently limit scalability, though techno-economic analyses suggest potential competitiveness in applications like electrolysis if durability under industrial conditions is proven. Future progress hinges on developing non-precious metal compositions and establishing cost-effective manufacturing and recycling pathways alongside continued fundamental research.

### 4.2. Transition Metal Compounds

While alloy catalysts leverage compositional tunability and conductive networks, transition metal compounds offer distinct advantages through non-metallic elements (P, S, Se) that actively participate in the catalytic cycle. Nanoporous transition metal compounds have attracted significant attention as efficient catalysts for the electrocatalytic HER in recent years. Including phosphides [36,117,118], sulfides [119,120], and selenides [121,122], exhibit catalytic performance approaching that of noble metals through precise compositional design and structural control. This section systematically discusses modification strategies and their applications in HER for various compound types, focusing on key mechanisms such as electronic structure regulation, morphology engineering, and interface optimization, with specific examples illustrating how these strategies work together to enhance catalytic performance.

#### 4.2.1. Phosphides

Transition metal phosphides (TMPs) have emerged as promising alternatives to precious-metal catalysts in the electrocatalytic HER due to their unique dual-site synergy and tunable electronic structures.

Two-dimensional nanoheterojunctions between homologous CoP and NiCoP nanosheets were constructed on a nitrogen-doped carbon (NC) matrix via sequential carbonization and phosphorization of a pre-synthesized Ni_1x_Co_x_–LDH@C_3_N_4_ precursor [123]. The high specific surface area of the C_3_N_4_ nanosheets not only facilitated the uniform dispersion of active components but also contributed carbon and nitrogen active sites to the composite. Well-defined nanoheterojunctions between CoP and NiCoP were achieved at higher Ni/Co ratios, with their synergistic effects reflected in the overall HER performance. The resulting CoP/NiCoP/NC hybrid exhibited outstanding HER activity and operational stability across a wide pH range, requiring overpotentials of only 75 mV in 1 M KOH, 60 mV in 0.5 M H_2_SO_4_, and 123 mV in 1 M PBS to achieve a current density of 10 mA cm^−2^. The remarkable HER performance can be attributed to the two-dimensional architecture, large surface area with abundant active sites, synergistic alloying and heterointerface effects between CoP and NiCoP, as well as electronic interactions between the phosphides and the NC support. Notably, the HER performance of the hybrid material is comparable or superior to that of recently reported earth-abundant electrocatalysts. Beyond high catalytic activity, this study provides valuable insights into the integration of multiple phases during synthesis and the influence of interfacial synergy on catalytic behavior. Given its high activity and stability over a broad pH range, the CoP/NiCoP/NC electrocatalyst shows potential for overall water splitting in extended pH environments when coupled with an efficient and stable oxygen evolution catalyst.

Cr-doped CoP nanorod arrays illustrate the multiple roles of heteroatom doping in modulating electronic properties and enhancing stability. Incorporation of Cr induces notable lattice strain and reconstructs the electronic coordination environment [124]. Density functional theory calculations reveal that hybridization between Cr 3d and Co 3d orbitals generates new electronic states near the Fermi level, effectively optimizing the binding energy of hydrogen intermediates. Moreover, Cr species preferentially migrate to the surface during catalysis, forming a stable Cr–O protective layer that kinetically suppresses over-oxidation and dissolution of active components. Electrochemical impedance spectroscopy measurements indicate a ≈60% reduction in charge transfer resistance after doping, confirming significantly improved electron transfer kinetics. Benefiting from this unique stabilization mechanism, the catalyst requires an overpotential of only 209 mV to reach 500 mA cm^−2^ and maintains stable operation for over 20 h.

**Figure 5:**
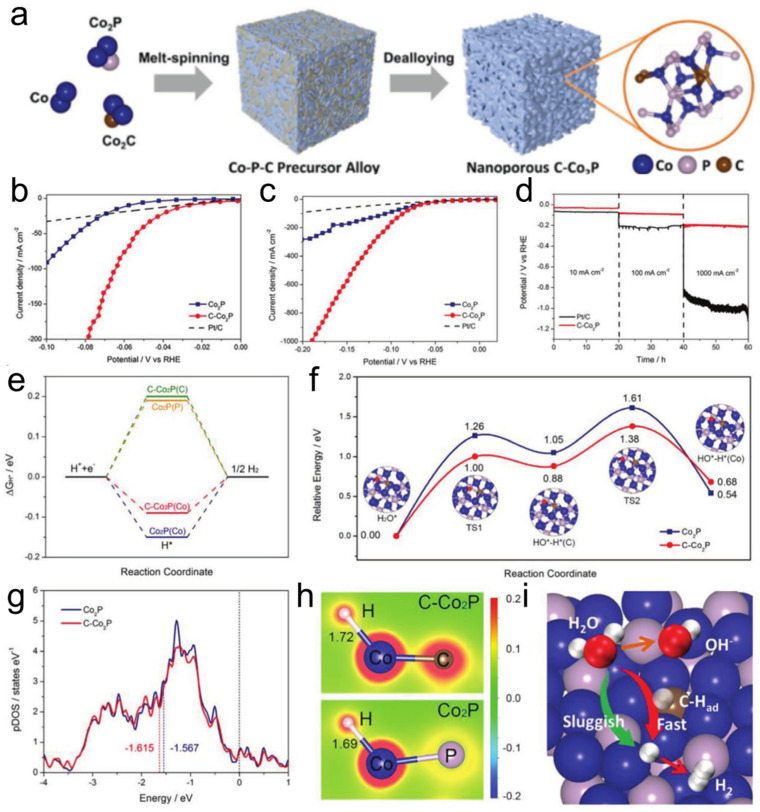
(**a**) Schematic of the preparation of nanoporous C-Co_2_P electrocatalysts. (**b**) Polarization curves measured in 1 M KOH. (**c**) Polarization curves in simulated alkaline seawater electrolyte, demonstrating high activity at industrial-level current densities. (**d**) Galvanostatic stability tests at various current densities, confirming outstanding operational durability over 60 h. (**e**) Computed hydrogen ΔG_H_* on Co sites. (**f**) Energy profiles for the water dissociation step via the proton-delivery pathway. (**g**) Projected density of states (pDOS) of Co atoms, showing the downshift in the d-band center after C doping. (**h**) Charge density difference plots illustrating electron redistribution. (**i**) Schematic diagram summarizing the proposed enhanced HER mechanism facilitated by carbon doping [125].

In a parallel strategy that employs targeted atomic doping to modulate electronic structure, Xu et al. demonstrated the efficacy of carbon incorporation in nanoporous cobalt phosphide (C-Co_2_P) for alkaline hydrogen evolution [125]. The catalyst was fabricated via a straightforward electrochemical dealloying process, which, as illustrated in Figure 5a, involved the selective dissolution of cobalt from a Co-P-C precursor alloy to create a bicontinuous, three-dimensional nanoporous skeleton. This unique architecture directly contributed to outstanding electrochemical performance, evidenced by polarization curves in Figure 5b where the optimal C-Co_2_P achieved an exceptionally low overpotential of 30 mV at 10 mA cm^−2^ in 1 M KOH, surpassing the activity of commercial Pt/C. Crucially, this high activity was maintained under industrially relevant conditions in simulated alkaline seawater, with Figure 5c showing that C-Co_2_P could deliver a large current density of 1000 mA cm^−2^ at an overpotential of only 192 mV. The binder-free electrode also exhibited exceptional durability, as Figure 5d confirmed negligible potential degradation over 60 h of operation at high current densities. To unravel the origin of this superior performance, DFT calculations were employed. These computations first established that carbon doping optimizes the ΔG_H_* at the Co sites to a near-ideal value of −0.09 eV (Figure 5e). Furthermore, they revealed a novel reaction pathway wherein the carbon dopant acts as a mediator, forming a C-H_ad_ intermediate that drastically reduces the energy barrier for the water dissociation step from 1.26 eV in Co_2_P to 1.00 eV in C-Co_2_P (Figure 5f). The electronic origin of this enhancement was traced to a downshift in the d-band center of Co atoms induced by the more electronegative carbon, as shown by the projected density of states in Figure 5g, which weakens the binding of reaction intermediates. This electron redistribution was visually confirmed by the charge density difference plots in Figure 5h. The collective mechanism, summarized schematically in Figure 5i, portrays the carbon dopant as a key facilitator that optimizes both water dissociation and hydrogen desorption.

#### 4.2.2. Sulfides

The catalytic performance of dichalcogenides is strongly influenced by their phase structure and defect concentration, a characteristic that distinguishes them from phosphide-based systems. Various strategies, including phase engineering, defect control, and morphology design, have been developed to enhance their electrocatalytic hydrogen evolution performance [126,127,128,129]. The following analysis examines these approaches in detail using specific research examples.

**Figure 6:**
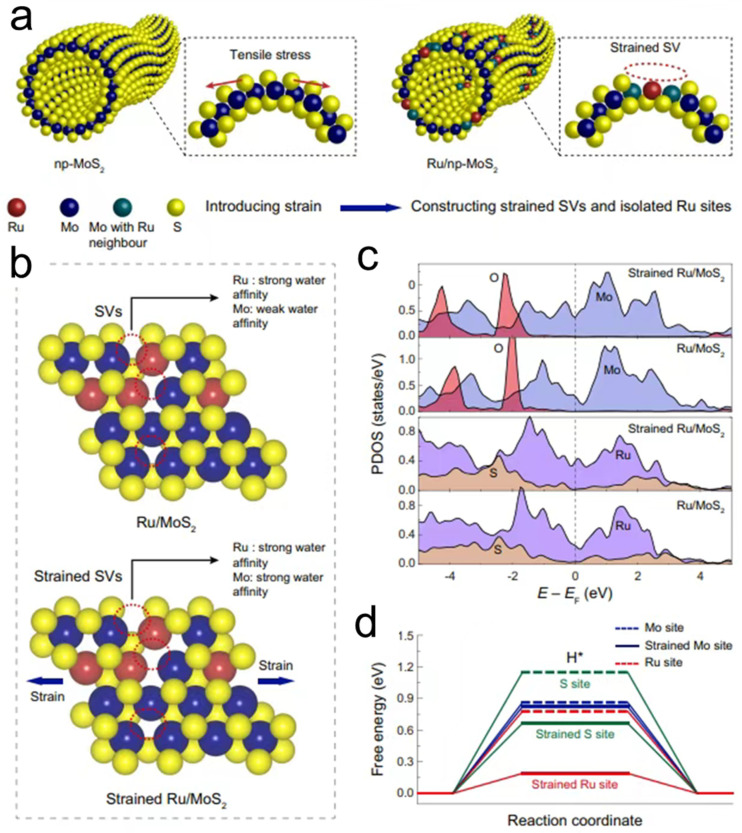
(**a**) Illustration of the construction Ru/np-MoS_2_. (**b**) Geometries of Ru/MoS_2_ before and after the applied strain. (**c**) Calculated PDOS of Ru/MoS_2_ before and after the applied strain. (**d**) Free energy diagrams for hydrogen adsorption at different sites [130].

In addressing key challenges in single-atom catalysts for the HER, including limited active site diversity and constrained reactant mass transfer, Jiang et al. developed a rational strain engineering strategy utilizing a nanoporous MoS_2_ substrate [130]. As illustrated in their study, the synthesis process (Figure 6a) involved anchoring ruthenium single atoms onto a three-dimensional bicontinuous MoS_2_ framework to construct the Ru/np-MoS_2_ catalytic system. Their theoretical calculations revealed that curvature-induced tensile strain simultaneously optimizes the electronic structures of ruthenium sites and adjacent sulfur vacancies. Specifically, Figure 6b demonstrates the atomic structural evolution under applied strain, while Figure 6c shows the enhanced density of d-orbital electronic states near the Fermi level of ruthenium sites. Furthermore, Figure 6d presents the optimized hydrogen adsorption free energy at different active sites under strain modulation. The strain engineering strategy transforms sulfur vacancies into efficient water molecule traps, significantly enhancing local reactant concentration at the interface. Concurrently, strain modulation substantially improves the catalytic activity of ruthenium sites by reducing the energy barrier for water dissociation and optimizing hydrogen intermediate adsorption. This strain-enhanced synergistic mechanism was verified through operando X-ray absorption spectroscopy and ambient-pressure X-ray photoelectron spectroscopy. The resulting catalyst exhibits exceptional alkaline HER performance, achieving a current density of 10 mA cm^−2^ at only 30 mV overpotential with a Tafel slope of 31 mV dec^−1^, significantly surpassing commercial platinum/carbon catalysts while maintaining excellent stability. Their methodology enabled the formation of atomic-scale cobalt arrays covalently bonded with the distorted MoS_2_ substrate, creating an interfacial catalyst system. This strategy demonstrates an alternative pathway for enhancing HER performance through precise control of single atom positioning and coordination environment. These works collectively demonstrate innovative approaches for overcoming performance limitations in single-atom catalysts through substrate engineering and atomic-scale design, providing valuable insights for developing advanced energy conversion catalysts.

The research by Jiang et al. [131] demonstrates the significant potential of integrating theoretical and experimental approaches. It not only reveals the critical role of single-atom catalysts in the HER but also provides a solid experimental foundation and theoretical basis for the rational design and optimization of future single-atom catalysts. By combining DFT predictions with operando XAS experimental validation, this work offers important theoretical and experimental support for understanding the mechanism of single-atom catalysts (SACs) in the HER. In this study, operando XAS technique was employed to observe changes in the electronic structure and local atomic configuration of the Pt/np-Co_0_._85_Se catalyst under actual electrolysis conditions. The results are consistent with the electronic effects and structural modulation mechanisms predicted by DFT, confirming the electronic activation of Co sites by single-atom Pt. Real-time monitoring of Co–Se bond evolution and Pt electronic state changes further validated the accuracy of the theoretical model. This process revealed that Pt doping electronically activates the basal Co atoms, promoting water adsorption and dissociation during the Volmer step, thereby significantly enhancing the hydrogen generation rate. More importantly, in terms of performance, the Pt/np-Co_0_._85_Se catalyst exhibits outstanding HER performance, achieving low overpotentials of 29.8 mV and 35.9 mV in acidic and alkaline media, respectively. It also demonstrates remarkable stability during long-term testing, confirming its practical applicability. Specifically, in the Volmer step of the HER, Pt doping significantly reduces the energy barrier and accelerates the reaction kinetics, a performance advantage effectively verified by operando XAS experiments.

**Figure 7:**
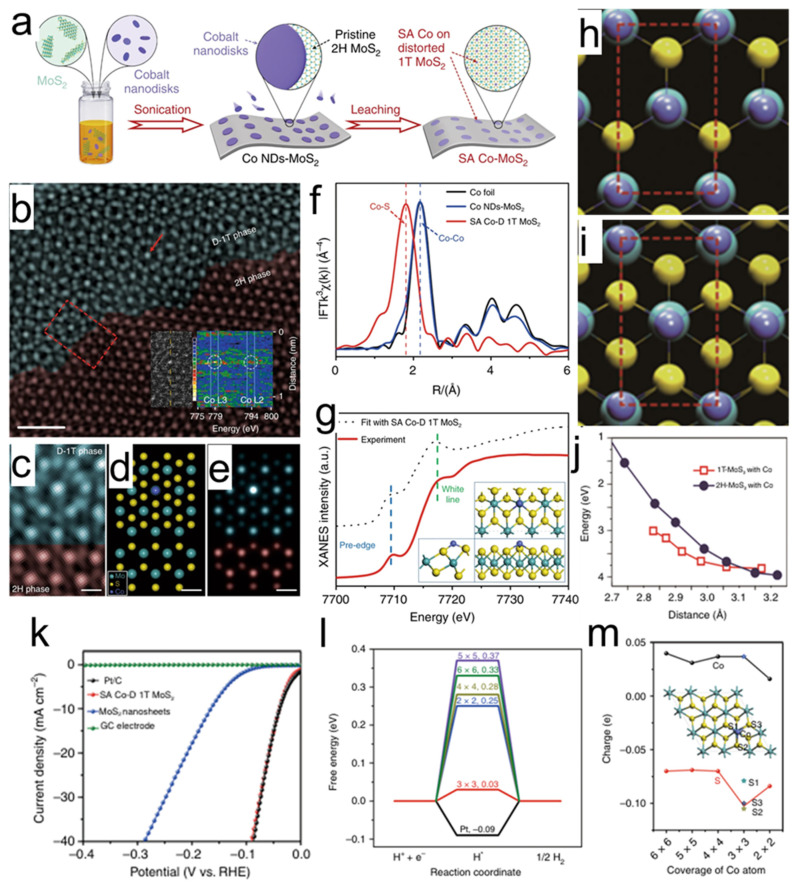
(**a**) Schematic diagram of the fabrication process for SA Co-D 1T MoS_2_; (**b**) Aberration-corrected HAADF-STEM image of SA Co-D 1T MoS_2_, showing the obvious junction between SA Co-D 1T MoS_2_ (dark cyan) and pristine 2H MoS_2_ (wine), The inset shows the HRTEM and EELS spectrum of SA Co-D 1T MoS_2_ (scale bar: 1nm); (**c**) Enlarged HAADF-STEM image in the red square area of b (scale bar: 2Å); (**d**) Theoretical model and (**e**) simulated STEM images using QSTEM simulation software (scale bar: 2Å). (**f**) FT-EXAFS spectra of SA Co-D 1T MoS_2_ and bulk cobalt foil at the Co K-edge. (**g**) Co K-edge XANES of SA Co-D 1T MoS_2_ and fitted curve. The inset shows the atomic structure of SA Co-D 1T MoS_2_; (**i**) 2H and (**h**) 1T atomic structures of MoS_2_ assembled with Co atomic layer calculated by first-principles. (**j**) Energies of 2H MoS_2_ and 1T MoS_2_ assembled with Co atomic layer as a function of Co–Co distance calculated by first-principles method based on the single layer 2H MoS_2_ and atomic Co as the reference state with the formula ΔE = E_2H-MS_ + E_Co_—E_MS-Co_; (**k**) Polarization curves of different catalysts tested in Ar-saturated 0.5M H_2_SO_4_; (**l**) Calculated free-energy diagram for HER at a potential of U = 0 relative to the standard hydrogen electrode at pH = 0 for different atomic Co loading amounts; (**m**) e the electron charge of Co and S adjacent to Co as a function of Co coverage [132].

The practical application of atomically dispersed metal catalysts still faces challenges such as difficulties in controlling atomic positions, limited loading density, and unclear interactions with the support. To address these issues, researchers led by Qi developed a synthesis strategy based on electrochemical cyclic voltammetry, successfully constructing a cobalt single-atom array on distorted 1T-phase MoS_2_ nanosheets (SA Co-D 1T MoS_2_) [132]. The preparation process of this material is shown in Figure 7a: first, cobalt nanodots and MoS_2_ nanosheets are combined via Co–S bonds through ultrasonic treatment to form a Co NDs/MoS_2_ precursor; subsequently, CV cycling treatment enables cobalt to be fixed in situ on the MoS_2_ surface in the form of single atoms, forming an orderly arranged cobalt single-atom array. The structural characterization results show that cobalt atoms are uniformly distributed on the D-1T MoS_2_ substrate. In the HAADF-STEM images in Figure 7b–e, clear cobalt atomic signals (indicated by red arrows) and the phase interface between SA Co-D 1T MoS_2_ and 2H MoS_2_ are visible. Electron energy loss spectroscopy further confirms the presence of cobalt, and Z-contrast imaging shows that cobalt atoms are located directly above the triangular molybdenum sites. The EXAFS spectrum in Figure 7f shows only a Co–S coordination peak (1.79 Å), indicating that cobalt is bonded to sulfur atoms in the form of single atoms. The XANES simulated structure in Figure 7g further reveals that cobalt atoms are located atop molybdenum atoms, coordinated with three sulfur atoms, forming a stable local structure. Theoretical calculations elucidate the origin of the structure’s stability and activity. Figure 7h–j show that the adsorption energy of cobalt on 1T MoS_2_ (4.37 eV/Co) is higher than that on the 2H phase (3.96 eV/Co), indicating that the 1T phase is more conducive to cobalt anchoring. Figure 7i demonstrates that the 1T structure exhibits superior stability when the Co–Co distance is less than 3.10 Å and the strain is approximately 3.70%. In the electrocatalytic hydrogen evolution reaction, SA Co-D 1T MoS_2_ exhibits excellent performance. Figure 7k show that in an acidic medium, its onset overpotential is only 42 mV, and the Tafel slope is 32 mV·dec^−1^, outperforming most non-noble metal catalysts and Pt/C, with almost no activity decay after 10,000 cycles. DFT calculations in Figure 7l,m further indicate that in a 3 × 3 superlattice with a cobalt coverage of 3.70%, the |ΔG_H_*| value is only 0.03 eV, close to the ideal value. The study reveals that the synergistic interaction between the cobalt single-atom array and the distorted 1T MoS_2_ is the key mechanism behind its high HER performance.

Guo et al. provided both theoretical and experimental evidence for the charge self-regulation effect in metastable 1T’’’-MoS_2_, which can be leveraged to modulate the electronic states of active sites and enhance HER performance [133]. Their findings indicate that increasing the concentration of S vacancies promotes the activation of Mo–Mo bonds, leading to a redistribution of electron density around adjacent S atoms and thereby optimizing hydrogen adsorption behavior. By employing a chemical etching strategy, they synthesized 1T’’’-MoS_2_-VS with tunable sulfur vacancy concentrations. Electrochemical measurements demonstrated that the optimized sample, 1T’’’-MoS_2_−10.6%, achieved an overpotential of 158 mV at 10 mA cm^−2^ and a Tafel slope of 74.5 mV dec^−1^, significantly surpassing the performance of defective 2H-MoS_2_-VS. These results confirm the critical role of charge self-regulation via activated Mo–Mo bonds in enhancing HER activity.

A series of transition metal sulfide electrocatalysts were successfully synthesized to investigate their surface reconstruction under high anodic potential and the corresponding impact on HER performance, with the aim of advancing practical applications [134]. The study systematically revealed two distinct surface reconstruction pathways in MoS_2_/NiS/CC during both high-potential anodic treatment and HER operation, quantitatively correlating the resulting sulfur/oxygen (S/O) species transformation with HER performance. It was found that the surface “melting” phenomenon and the evolving S/O ratio significantly influenced catalytic behavior. Moderate oxygen incorporation was shown to optimize HER kinetics, whereas excessive oxygen content impaired both intrinsic activity and electrical conductivity. Through controlled in situ reconstruction, HER performance was precisely regulated, with 20 s anodic treatment identified as optimal. The resulting 20 s-NMSO electrode required only 155.6 mV overpotential to reach 200 mA cm^−2^, approaching the performance of commercial Pt/C (151.4 mV), and even outperformed Pt/C at higher current densities by delivering 1 A cm^−2^ at an ultra-low overpotential of 231 mV. Furthermore, the catalyst demonstrated exceptional stability with merely 0.06% voltage increase after 144 h of continuous operation. The validity of this anodic oxidation strategy was further verified in CoS/NiS materials, confirming its general applicability and providing new insights for developing advanced transition metal based HER electrocatalysts.

#### 4.2.3. Selenides

The optimization strategies for the catalytic performance of selenides differ notably from those for sulfide systems, placing greater emphasis on the synergistic regulation of alloying and interface engineering. The construction of heterogeneous interfaces, precise control of alloy composition, and the design of hierarchical porous structures can effectively enhance their electrocatalytic hydrogen evolution performance [135].

Wen et al. constructed a black phosphorus nanosheet/CoNiSe_2_ nanoflower heterojunction (BP/CoNiSe_2_) via a one-step solvothermal approach [122]. CoNiSe_2_ nanoflowers were in situ grown on exfoliated BP nanosheets to form a unique 2D/3D heterostructure. This interfacial coupling is stabilized by the formation of P–Ni/Co and P–Se covalent bonds, as confirmed by XPS analysis. Crucially, a directional built-in electric field (BIEF) was established at the interface due to the work function difference between BP (4.51 eV) and CoNiSe_2_ (5.17 eV), driving electron transfer from BP to CoNiSe_2_. Density functional theory calculations revealed that this BIEF induces a downshift in the d-band centers of Co/Ni dual-active sites, which optimizes ΔG_H_* closer to the ideal value, thereby accelerating the hydrogen adsorption/desorption kinetics. Electrocatalytic evaluation demonstrated that the optimal BP/CoNiSe_2_ heterojunction achieves an exceptionally low overpotential of 82 mV at 10 mA cm^−2^ and a Tafel slope of 64 mV dec^−1^ in alkaline medium, rivaling commercial Pt/C. When applied as a cathode in an anion exchange membrane water electrolyzer, it delivered a high current density of 350 mA cm^−2^ at 1.79 V with outstanding operational stability exceeding 320 h. This work exemplifies a rational interface engineering strategy for enhancing electrocatalytic performance through built-in electric field-mediated electronic structure modulation.

**Figure 8:**
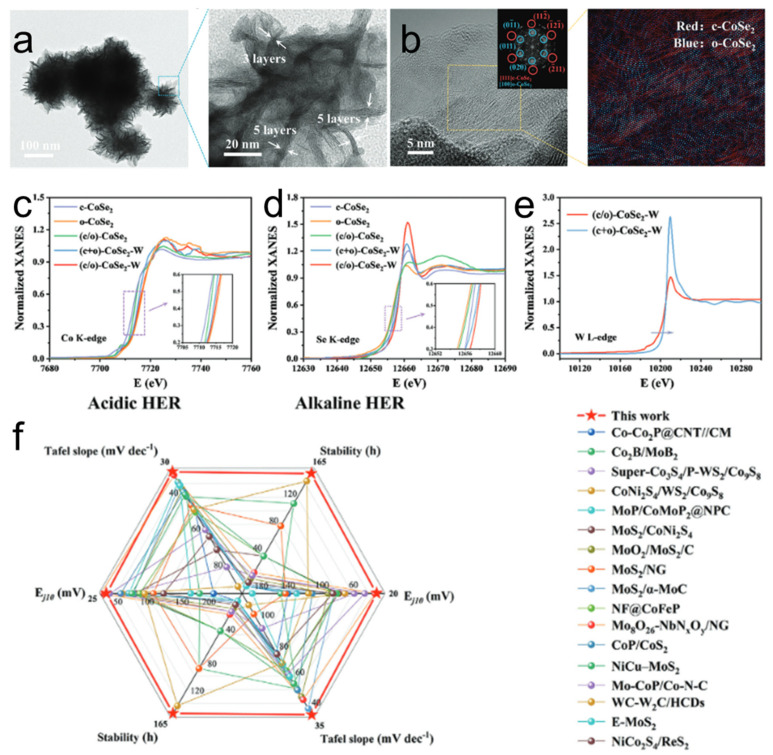
(**a**) TEM images of (c/o)-CoSe_2_-W. (**b**) High-resolution TEM image of the cross-section of (c/o)-CoSe_2_-W. The inset gives the corresponding FFT pattern within the yellow box, which displays two sets of patterns from the [111] zone axis of c-CoSe_2_ and [100] zone axis of o-CoSe_2_ and Atomic-resolution image. (**c**) Normalized Co K-edge XANES. (**d**) Normalized Se K-edge XANES. (**e**) Normalized W L-edge XANES. (**f**) Comprehensive comparison of the catalytic performance of state-of-the-art nonprecious metal catalysts reported in the literature with the catalyst in this work.

Zhang et al. [136] developed a high-performance electrocatalyst by incorporating tungsten into cobalt diselenide to create a mixed-phase interface, which enables exceptional HER activity in both acidic and alkaline electrolytes (Figure 8a). This study presents a W-doped CoSe_2_ electrocatalyst featuring a mixed cubic/orthorhombic phase interface ((c/o)-CoSe_2_–W) for the HER. As shown in Figure 8b, the incorporation of W induces a phase transition, creating a well-defined interface as directly evidenced by HR-TEM and FFT analysis, which confirms the coexistence of cubic CoSe_2_ ([111] zone axis) and orthorhombic CoSe_2_ ([100] zone axis) within a nanoflower-like morphology. XANES analysis reveals that this interfacial structure leads to a significant electronic redistribution: shifts in the Co and Se K-edges to higher energies indicate electron depletion, while a decreased white-line intensity at the W L_3_-edge signifies electron accumulation at the W sites (Figure 8c–e). As shown in Figure 8f, this electronic modulation, facilitated by the Co–Se–W bridging structure, optimizes the adsorption energetics of reaction intermediates. As a result, the (c/o)-CoSe_2_–W catalyst achieves exceptional HER activity, with record-low overpotentials of 29.8 mV in alkaline and 35.9 mV in acidic media among non-precious metal catalysts, alongside excellent stability. This work underscores the critical role of synergistic phase-interfacial doping in developing high-performance and durable electrocatalysts.

Shen et al. developed a crystalline-amorphous CoSe_2_/CoP heterojunction through controlled phosphorization of ultrathin Co_0_._85_Se nanosheets. Advanced electron microscopy and spectroscopic analyses confirmed the coexistence of crystalline orthorhombic CoSe_2_ and amorphous CoP phases with well-defined interfaces. The strong electronic coupling at these interfaces induces significant charge redistribution, leading to an elevated valence state of Co and a downshift in the d-band center. This electronic optimization weakens hydrogen adsorption strength, as verified by density functional theory calculations showing near-optimal ΔG_H_* of −0.107 eV. The catalyst demonstrates exceptional HER performance across pH-universal conditions, achieving low overpotentials of 65 mV in acidic, 151 mV in alkaline, and 185 mV in neutral media at 10 mA cm^−2^, significantly outperforming its crystalline-crystalline counterpart. The amorphous component further enhances the electrochemically active surface area and strengthens interfacial electronic coupling, collectively contributing to the superior catalytic activity. This work establishes crystalline-amorphous interface engineering as an effective strategy for optimizing transition metal compound electrocatalysts.

## 5. Conclusions and Outlook

This review systematically summarizes recent advances in nanoporous electrocatalysts for the HER, covering both alloy systems and compound materials. Alloy-based nanoporous electrocatalysts, characterized by their three-dimensional interconnected porous networks, facilitate rapid mass and electron transport while providing abundant active sites. These structural advantages endow them with exceptional electrocatalytic performance, making them well-suited for hydrogen production via electrochemical water splitting. Key attributes of these nanomaterials—including alloying effects, unsaturated coordination, and surface strain—prove highly effective in optimizing both the thermodynamic and kinetic aspects of the hydrogen evolution reaction. Table 1 lists a comparison of the electrochemical performance of some representative nanoporous electrocatalysts by benchmarking overpotential at 10 mA cm^−2^, Tafel slope and stability. Over the past decade, significant progress has been made in the development of nanoporous alloy systems, encompassing bimetallic alloys, ordered intermetallic, high-entropy alloys, and related transition-metal-based compounds. 

In terms of material design, multi-element synergistic strategies have proven to be effective approaches for enhancing catalyst performance. Precise regulation of alloy composition and crystal structure enables optimization of the electronic structure of active sites, achieving accurate control of hydrogen adsorption free energy. Second, the construction of nanoporous structures significantly improves catalyst performance. The three-dimensional interconnected pore channels not only provide abundant active sites but also facilitate mass transport of reactants and products, enabling excellent performance even at high current densities. In interface engineering, the construction of heterogeneous structures optimizes reaction pathways through interfacial electronic effects, thereby improving catalytic efficiency. Particularly noteworthy is that the introduction of defect engineering provides new avenues for enhancing catalyst performance. These defects not only create new active sites but also modulate surface electronic structure through strain effects. Furthermore, transition metal compounds exhibit distinct characteristics: active site engineering in sulfides, metal-like conductivity in phosphides, defect regulation in oxides, and multi-element doping strategies in selenides all offer diverse options for catalyst design in different application scenarios. In spite of the substantial progress in design/synthesis and property investigation of nanoporous electrocatalysts, there remain great challenges in further development of electrochemical water splitting.

While the nanoporous catalysts reviewed herein exhibit promising hydrogen evolution reaction performance at the laboratory scale, their translation to industrial electrolyzers remains challenging and requires a systematic evaluation of scalability, economic viability, and long-term stability. From a synthesis perspective, no single strategy excels universally. Electrodeposition demonstrates notable compatibility with scale-up requirements due to its ambient conditions and adaptability to continuous manufacturing. In contrast, dealloying, despite yielding well-defined porous architectures, faces challenges related to precursor cost and the handling of corrosive media. Other methods, such as hydrothermal/solvothermal synthesis and laser-based processing, are constrained by their batch-processing nature, high energy inputs, and limited throughput, posing significant techno-economic barriers. Beyond synthesis, the operational stability under industrial conditions is paramount and is threatened by several intrinsic degradation mechanisms: (1) Structural coarsening driven by the high surface energy of nanoscale ligaments, leading to active surface area loss; (2) Mechanical fatigue from vigorous gas bubble evolution, potentially causing ligament fracture or active particle detachment; and (3) Surface poisoning by electrolyte impurities coupled with chemical degradation via oxidation (in alkaline media) or dissolution (in acidic media). The industrial electrolyte environment itself, with its specific composition and impurities, profoundly influences these degradation pathways and will ultimately dictate the catalyst’s lifetime.

The electrolyte composition critically dictates both the activity and stability of nanoporous electrocatalysts for the hydrogen evolution reaction. Kinetically, buffering anions such as phosphate and carbonate enhance the rate-determining water dissociation step through a proton-relay mechanism. Their moderate adsorption can also optimize the hydrogen binding energy. However, excessive adsorption blocks active sites, and parasitic reactions including carbonate decomposition may occur. Regarding stability, the electrolyte influences performance through chemical corrosion, surface reconstruction, and product management. While certain anions like phosphate can form protective surface layers, aggressive ions such as chloride may trigger pitting corrosion. The high surface area of nanoporous structures intensifies these challenges. Bubble accumulation from gas evolution can mechanically damage the porous framework, and the leaching of active components disrupts the precisely engineered surface chemistry.

Electrolyte engineering and catalyst design should be optimized in tandem, rather than considered in isolation, in the context of the HER. The performance of catalysts is not solely determined by their intrinsic material properties but is also significantly influenced by factors such as electrolyte composition, ion types, concentration, buffering capacity, solvent properties, and impurities. By fine-tuning the electrolyte composition, it is possible to enhance proton or hydrogen intermediate availability, improve the water/ion structure at the electrode-electrolyte interface, increase proton transfer efficiency, reduce side reactions, and improve catalyst stability. For instance, phosphate or carbonate-based buffering systems can maintain stable pH values and promote efficient proton and hydrogen intermediate exchange, although they may interact with the catalyst metal, potentially leading to coordination or precipitation reactions. Therefore, selecting an appropriate electrolyte requires careful consideration of the catalyst’s properties to ensure compatibility. Optimizing the electrolyte can prevent unnecessary surface passivation or the formation of side products on the catalyst while maintaining system efficiency.

Looking forward, machine learning (ML) presents opportunities to complement conventional methodologies in advancing nanoporous HER electrocatalysts. The integration of data-driven approaches with physicochemical models may enable systematic decoding of the complex relationships among compositional design, hierarchical porous architectures, and catalytic properties. Emerging evidence suggests that coupling active learning frameworks with multi-objective optimization algorithms could guide the design of catalysts with optimized porous structures while balancing active site density and mass transport efficiency. Nevertheless, several challenges require resolution before full realization of this potential: the systematic construction of high-quality datasets, enhancement of model interpretability, and establishment of robust feedback loops between computational predictions and experimental validation. With continued interdisciplinary collaboration, such integrated approaches may offer novel design paradigms for high-performance catalysts tailored to industrial electrolyzers, though their practical efficacy must be rigorously confirmed through experimental verification and long-term stability assessment.

## Figures and Tables

**Table 1:** List of the electrocatalytic activities of nanoporous catalysts for the HER.

Catalyst	Overpotential at 10 mA/cm^2^	Tafel Slope (mV/dec)	Electrolyte	Stability	Reference
NiMoB	31 mV	73	1 M KOH	100 h	[35]
Ni-Mo	31 mV	39.5	1 M KOH and 1 M PBS	20 h	[87]
NiCo(OH)x-CoyW	21 mV	35	1 M KOH	20 h	[137]
Ni-Mo and NiMo	24 mV	37	1 M KOH	48 h	[77]
NiCuMo	63 mV	36	1 M KOH	20 h	[78]
	115 mV	115	1 M PBS		
NiCo(OH)x-CoyW	21 mV	35	1 M KOH	20 h	[138]
NiFeCoCuTi	134 mV at 1 A/cm^2^	43	1 M KOH	240 h	[70]
Ni/CeOx	201 mV	41	Alkaline seawater	100 h	[85]
NiFe HEA	188 mV		1 M KOH	100 h	[84]
3D Porous Ni-P/Ni	108 mV	75	1 M KOH	20 h	[83]
MoO_2_-FeP	103 mV	42	1 M KOH	24 h	[139]
NiFeMo	15 mV	73	1 M KOH	100 h	[81]
NiMo/NiMoS	38 mV	38	1 M KOH	100 h	[120]
NiMoN	130 mV at 1 A/cm^2^	28.3	1 M KOH	100 h	[140]
Co@CoO/RuO_2_	198 mV	57.1	1 M KOH	50 h	[141]
CoSe_2_	112 mV	36	0.5 M H_2_SO_4_	15,000 s	[142]
Mg_2_Ni/MgNi_2_	69.2 mV	37.39	1 M KOH	38 h	[92]
Mo_2_C/MoNi_4_	49.5 mV	44.35	1 M KOH	100 h	[93]
Fe_2_Mo	200 mV	71	1 M KOH	400+ h	[90]
MoNiS_3−x_O_x_	155.6 mV	65.52	1 M KOH	144 h	[134]
Fe-MoS_2_/rGO	197 mV	53	1 M KOH	12 h	[143]
Cr-CoP	47 mV	46	0.5 M H_2_SO_4_	30 h	[124]
	131 mV	67	1 M PBS		
	67 mV	31	1 M KOH		
CoP/NiCoP	75 mV	64	1 M KOH	3000 cycles	[123]
	60 mV	58	0.5 M H_2_SO_4_		
	123 mV	78	1 M PBS		
PtSe_2_/PtCo	38 mV	22	0.5 M H_2_SO_4_	1000 cycles	[144]
BP/CoNiSe_2_	82 mV	64	1 M KOH	320 h	[122]
Ni_3_Se_4_−Ni_3_N	60 mV	51.1	1 M KOH	100 h	[145]
Ru-MoS_2_	30 mV	31	1 M KOH	40 h	[130]
Pt_2_Sn_2_S_6_	13 mV	34	1 M KOH	100 h	[146]
AC-NP-CuNiCo	1.53 V for OWS	50	1 M KOH	100 h	[110]
CoSe_2_/CoP	65 mV	54	0.5 M H_2_SO_4_	50 h	[147]
BP/CoNiSe_2_	82 mV	64	1 M KOH	320 h	[122]
Co-1T MoS_2_	42 mV	32	1 M KOH	240 h	[132]
Pt/np-Co_0_._85_Se	55 mV	35	1.0 M PBS	40h	[131]
MoS_2_@NPG	70 mV	38	1 M KOH	1000 cycles	[148]
W-CoSe_2_	29.8 mV	36.2	1 M KOH	160 h	[136]
	35.9 mV	30.9	0.5 M H_2_SO_4_		
PdIr	5 mV	24	0.5 M H_2_SO_4_		[104]
FeCoNiNbPt Alloy	41 mV	87.2	0.5 M H_2_SO_4_	2200 + h	[116]
FeCoNiNbPt HEA	137 mV at 1 A/cm^2^	39.3	0.5 M H_2_SO_4_	Over 1,000,000 cycles	[103]
Au and Pt	40 mV (Pt)	100	Bicarbonate buffer		[149]
NiCoP	48 mV	32	1 M KOH	5000 cycles	[150]

List of the electrocatalytic activities of nanoporous catalysts for the HER.

## Data Availability

No new data were created or analyzed in this study.
